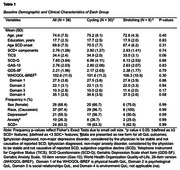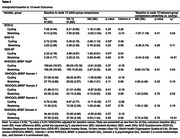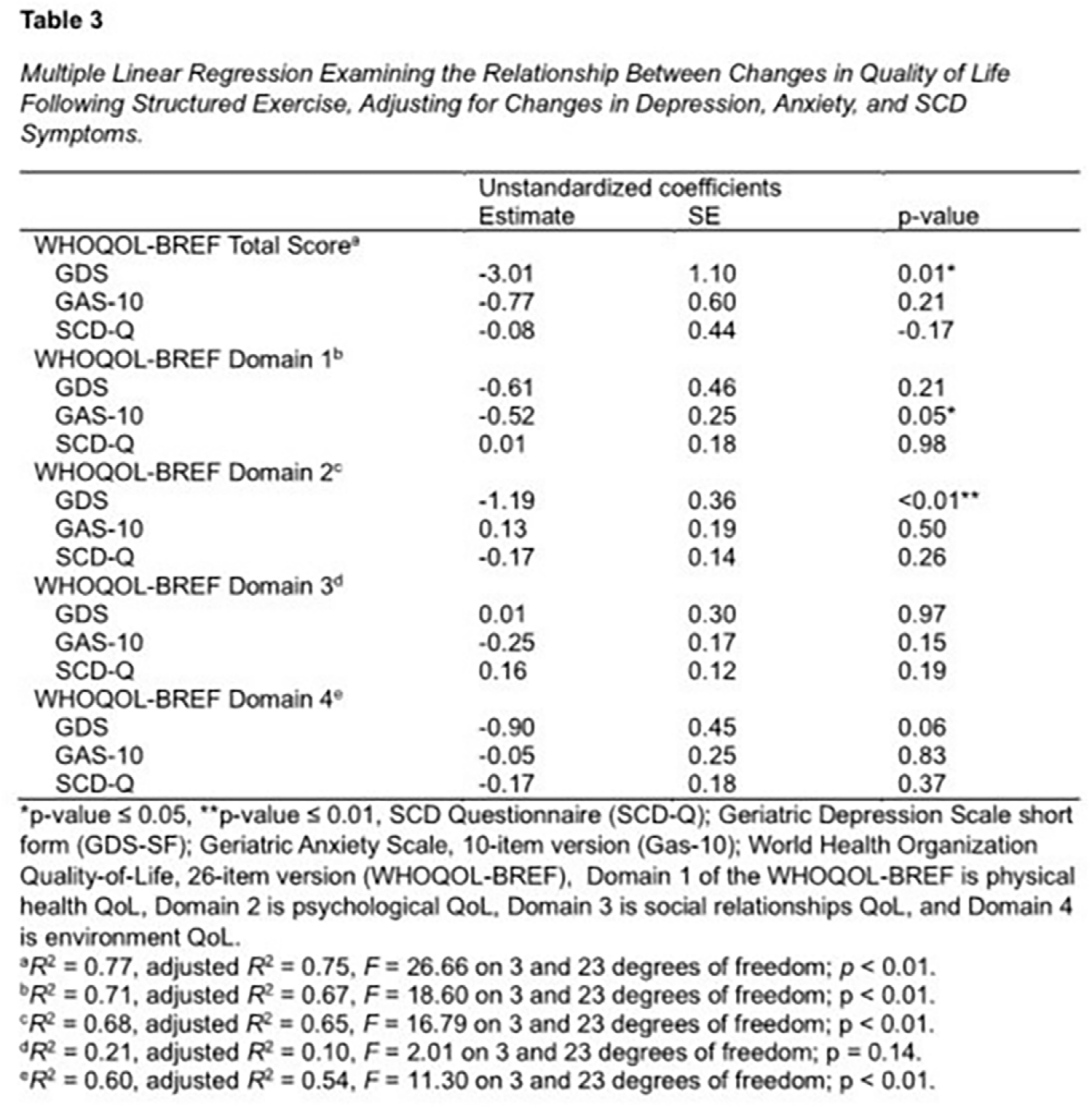# Impact of Aerobic Cycling on Affective Symptoms and Quality of Life in Persons Experiencing Subjective Cognitive Decline

**DOI:** 10.1002/alz.095430

**Published:** 2025-01-09

**Authors:** Rebecca Ann‐Maria Watry, Niloufar Hadidi, Mary Jo Kreitzer, Rozina Bhimani, Fang Yu, Dereck L. Salisbury

**Affiliations:** ^1^ University of Minnesota, Minneapolis, MN USA; ^2^ Arizona State University, Phoenix, AZ USA

## Abstract

**Background:**

Transition to subjective cognitive decline (SCD), and dementia is often accompanied by increased affective symptoms (i.e., anxiety and depression), which increase the risk of Alzheimer’s dementia and decreased quality of life (QoL). The hazard ratio of SCD with affective symptoms is significantly worse in comparison to SCD only. A paucity of research investigates the impact of treatments, including aerobic exercise, on SCD, affective symptoms, and QoL. The purpose was to examine the preliminary efficacy of moderate‐intensity cycling exercise versus control on SCD, affective symptoms, and overall QoL, including QoL domains.

**Methods:**

The Exergames Study randomized community‐dwelling older adults with SCD (n = 38) to 12 weeks (36 sessions) of exergames, cycling only, and stretching control groups using a 2:1:1 allocation ratio. For this secondary data analysis, the exergames and cycling groups were combined (n = 30). SCD symptoms, affective symptoms, and QoL were assessed by the SCD‐Q, GAS‐10, GDS‐SF, and WHOQOL‐BREF. Between‐group change was evaluated by ANCOVA, and relationships between changes in QoL were calculated with multiple linear regression, adjusting for baseline values.

**Results:**

Enrollment was from November 2019 ‐ December 2021. The mean age was 74.6 (7.4) years old and 69% were female (Table 1). There were no significant between‐group changes (Table 2). Large and moderate between‐group effect sizes were seen in favor of the stretching group for WHOQOL‐BREF Social domain (between group change = 1.90 [1.19], ηp2 = 0.19); GAS‐10 (between group change = ‐5.36 [0.29], ηp2 = 0.11); and WHOQOL‐BREF Physical Domain (between group change = 4.56 [4.40], ηp2 = 0.10). Increased affective symptom scores by one item corresponded with a decrease in predictive QoL scores (anxiety‐physical health ‐0.52 [0.25], p = 0.05; depressive‐overall ‐3.01 [1.10], p = 0.01; depressive‐psychological ‐1.19 [0.36], p < 0.01; Table 3).

**Conclusion:**

Although non‐significant, between‐group changes favored the control group. Changes in affective symptoms predicted changes in QoL. The participation attention received by this secluded population of older adults and COVID‐19 losses likely impacted results. Further research with larger groups (that does not span a pandemic) is necessary to elucidate the full impacts of aerobic cycling on SCD symptoms, affective symptoms, and QoL.